# Aetiology of diarrhoeal disease and evaluation of viral–bacterial coinfection in children under 5 years old in China: a matched case–control study

**DOI:** 10.1016/j.cmi.2015.12.018

**Published:** 2016-04

**Authors:** L.L. Li, N. Liu, E.M. Humphries, J.M. Yu, S. Li, B.R. Lindsay, O.C. Stine, Z.J. Duan

**Affiliations:** 1) National Institute for Viral Disease Control and Prevention, China CDC, Beijing, China; 2) University of Maryland, School of Medicine, Baltimore, MD, USA; 3) Merck & Co. Inc., North Wales, PA, USA

**Keywords:** Aetiology, bacteria, diarrhoea, quantitative PCR, virus

## Abstract

Globally, diarrhoeal diseases are the second leading cause of death among children under 5 years old. Few case–control studies on the aetiology of diarrhoea have been conducted in China. A case–control study on 922 children under 5 years old who presented with diarrhoea and individually matched controls was conducted in China between May 2011 and January 2013. Quantitative PCR was used to analyze stool samples for 10 diarrhoeal pathogens. Potential enteric pathogens were detected in 377 (81.8%) of 461 children with diarrhoea and 215 controls (46.6%, p <0.001). Rotavirus, norovirus GII, *Shigella* and adenovirus were qualitatively associated with diarrhoea. Using receiver operating characteristic curves, the optimal cutoff threshold for defining a symptomatic individual was 72, 5840, and 10^4^ copies per reaction for rotavirus (odds ratio 259), norovirus GII (odds ratio 10.6) and *Shigella* (odds ratio 5.1). The attributable fractions were 0.18 for rotavirus, 0.08 for norovirus GII, 0.01 for *Shigella* and 0.04 for adenovirus. Coinfections between pathogens were common. Two pairs, rotavirus and adenovirus, and norovirus GII and *Salmonella* were positively associated. The co-occurrence of rotavirus and sapovirus, astrovirus, enterotoxigenic *Escherichia coli* or *Campylobacter jejuni* only occurred in children with disease. Coinfection was not correlated with clinical symptoms. Quantitative data are critical. Our results indicate that increased pathogen loads increase the OR between diarrhoea and rotavirus, norovirus GII and *Shigella.* Coinfections with rotavirus and norovirus GII are common and occur in a nonrandom distribution. Despite testing for ten diarrhoeal pathogens, over two-thirds of cases do not have a recognized attributable cause.

## Introduction

Diarrhoeal disease accounts for the deaths of one in nine children worldwide; it is also responsible for 0.75 million deaths every year [1]. Diarrhoea alone amounts to an estimated 4.1% of the total global burden of disease [2] and is caused by a variety of enteric viruses, bacterial pathogens and parasites. The possible pathogenic culprits include rotavirus A, norovirus GI and GII, adenovirus, sapovirus, astrovirus, *Salmonella, Campylobacter jejuni, Shigella* spp. and enterotoxigenic *Escherichia coli* (ETEC) [3], [4]. The prevalence of these diarrhoea-causing agents differs according to demographic, socioeconomic, environmental hygiene and geographic factors. A number of previous epidemiologic studies have described the distribution of a variety of enteropathogens in south Asia, Africa, Europe, the Middle East, South America and the United States [5], [6], [7], [8], [9], [10], [11], [12]. However, none of these studies included China, and enteropathogen estimates in China are limited. Additionally, most of the previous studies focused on only a small number of diarrhoea-causing pathogens, and many did not include healthy controls.

Coinfection with multiple pathogens may be common among children with diarrhoea and may cause more severe diarrhoea than infection with a single pathogen [12], [13]. It is also important to include healthy controls in order to compare the distribution of exposure in healthy controls compared to cases [6], [10]. Previous case–control and longitudinal studies have identified and quantified multiple pathogens, including bacteria and viruses, using quantitative PCR (qPCR) [11], [14]. Their results suggested that assessing pathogen loads may improve the identification of pathogens causing diarrhoea in children, particularly for pathogens that are frequently present in both symptomatic and healthy children [7], [8], [11], [14]. There are few studies about interactions between virus and bacteria as a result of lack of asymptomatic controls, so little is known about the role of interaction between multiple pathogens in causing diarrhoea.

In this study, inpatient cases were matched to community controls to estimate the prevalence and pathogenicity of viruses and bacteria in children, both with and without diarrhoea, under 5 years of age in Northern and Southern China between 2011 and 2012.

## Methods

### Case and control definition

Diarrhoea was defined using the World Health Organization guidelines: the presence of three or more liquid stools in a 24-hour period. All cases were hospitalized with a primary diagnosis of diarrhoea and were thus considered severe. An asymptomatic control was defined by the lack of diarrhoea symptoms for a week before enrollment. Controls were matched by sex, age group (0–5, 6–11, 12–23, 24–35, 36–47 and 48–59 months) and location of residence. The time between enrollment of the case and its matched control was no more than 14 days.

### Setting, collection of faecal samples and study design

**Fig. 1:**
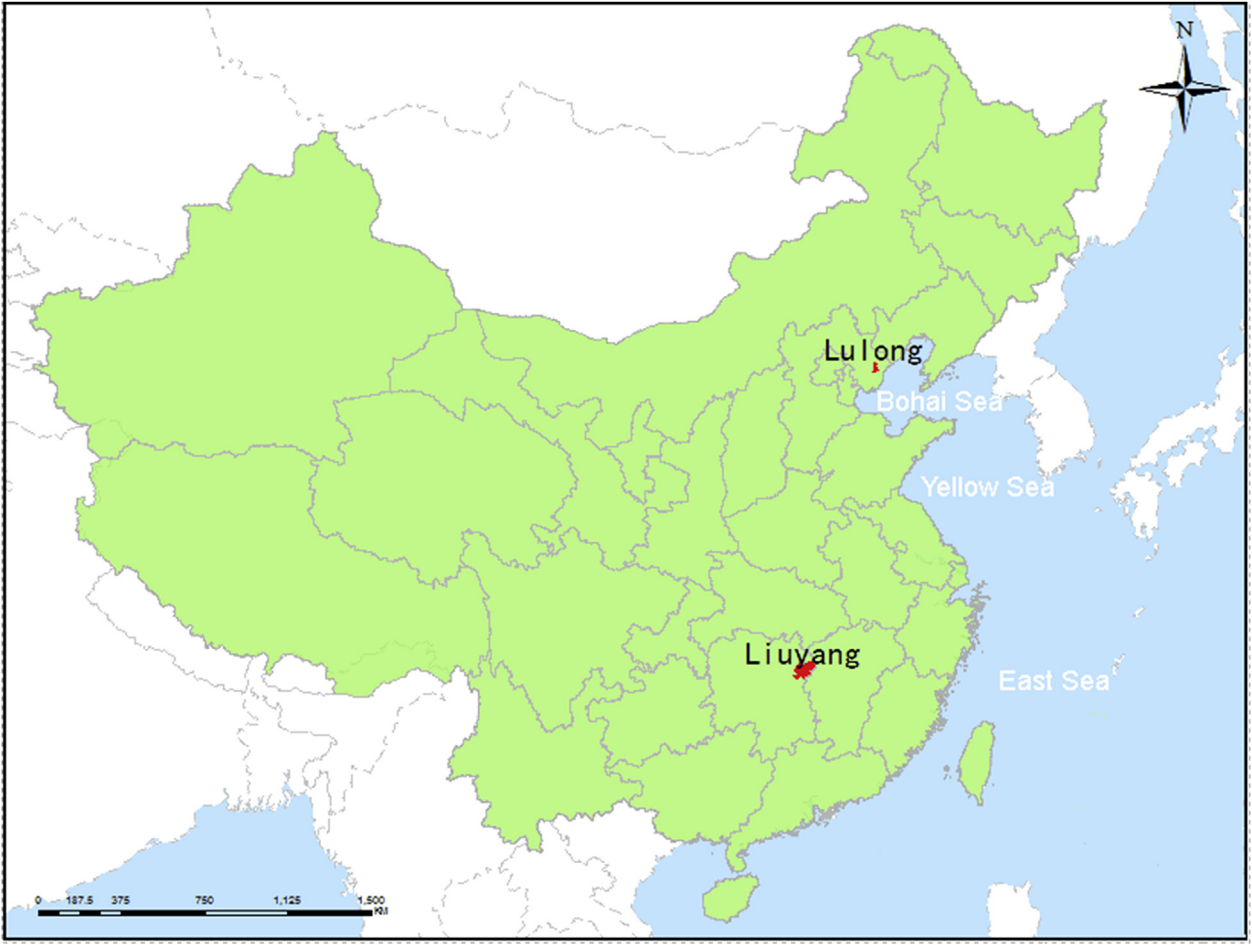
Location of study sites in China.

The study was carried out in two rural areas, one in northern China (Lulong, Hebei province) and the other in southern China (Liuyang, Hunan province) (Fig. 1). Between May 2011 and January 2013, diarrhoea samples were collected consecutively from inpatient children younger than 5 years of age at Women and Children's Hospital in Liuyang and People's Hospital of Lulong County and Women and Children's Hospital in Lulong. National health registry data were used for matching children. The control samples matched by sex, age and location were enrolled by a local health worker by visiting children without diarrhoea at home.

Demographic information (age and sex) and clinical symptoms (fever, vomiting and dehydration) were recorded for each child using a standardized questionnaire. Stool samples were collected by trained healthcare personnel using sterile stool containers and then stored at −20°C until further analysis.

### Laboratory procedures

Virus RNA and DNA were extracted from 200 μL of 10% faecal suspension in phosphate-buffered saline with use of the QIAamp MiniElute Virus spin kit (Qiagen, Germantown, MD, USA), according to the manufacturer's instructions. QIAamp DNA stool Mini kit (Qiagen) was used for genomic DNA extraction directly from 200 μL of 10% stool suspension. The nucleic acids were eluted in 100 μL volume, and 4 μL of this was used for qPCR. Rotavirus, norovirus GII, norovirus GI, sapovirus, astrovirus, adenovirus and *Salmonella* were detected using TaqMan probes for qPCR; *Shigella, C. jejuni,* and ETEC were detected using SYBR Green for qPCR. The primers/probes and the procedures for each reaction are listed in Table 1 and have been described previously [15], [16], [17], [18], [19], [20], [21], [22].

Amplification was performed in an ABI 7500 instrument (ABI, Foster City, CA, USA) in 20 μL reactions using the AgPath-ID One-Step RT-PCR Kit (ABI, for RNA targets), Universal Master mix (ABI, for DNA targets of TaqMan) and SYBR Green PCR Master Mix (ABI, for bacteria DNA targets) according to the manufacturer's instructions. The number of copies in each qPCR was calculated from standard curves of serial dilutions of *in vitro* transcription targets carrying synthetic target inserts. Standard curves were generated by plotting the log of the starting quantity of *in vitro* transcription targets RNA against the cycle threshold (C_T_) value obtained from the amplification of each dilution. The quality of qPCR products was judged from the slope and the correlation coefficient (*r*^2^) of standard curves.

### Statistical analysis

Chi-square statistical significance testing was performed to compare the difference in proportions of samples that were positive or negative by qPCR; mean copy numbers were compared by independent two-tailed *t* tests. We also performed logistic regression to describe the association between pathogen detection and diarrhoea. To examine the association between pathogen load and clinical symptoms, we calculated odds ratios (ORs) for every pathogen for every tenfold increase in copy numbers, starting at the 10^0^ copy of detection and using negative results as the reference group.

To estimate a clinically meaningful cutoff value of diarrhoeal pathogens, we constructed receiver operating characteristic (ROC) curves from the continuous measurement of the number of gene copies per sample by plotting the estimated sensitivity by 1-specificity. For the ROC analysis model, we included gene copies as the independent variable and case status as the outcome and dependent variable. The optimal cutoff value determined by the maximum Youden index: (J = max(sensitivity + specificity) − 1). A p value of <0.01 was considered statistically significant. All statistical analyses were done in SPSS 19 (IBM, Armonk, NY, USA).

The attributable fraction was calculated from the formula 1 − (1/OR)p, where p is the proportion of cases with the pathogen. The total attributable fraction equals 1 − (AF_rotavirus_)(1 − AF_norovirusGII_)(1 − AF_adenovirus_)(1 − AF_*Shigella*_) [23].

## Results

### Patients and samples

During the 20-month case–control study period, we enrolled a total of 461 cases and 461 controls. There were 239 pairs from Northern China (Lulong, Hebei province) and 222 pairs from Southern China (Liuyang, Hunan province) (Fig. 1). The majority of the children were between 1 and 24 months old (94% of cases and 93% of controls), with a median age of 10 months in both groups (Table 2).

### Occurrence of enteric pathogens

**Fig. 2:**
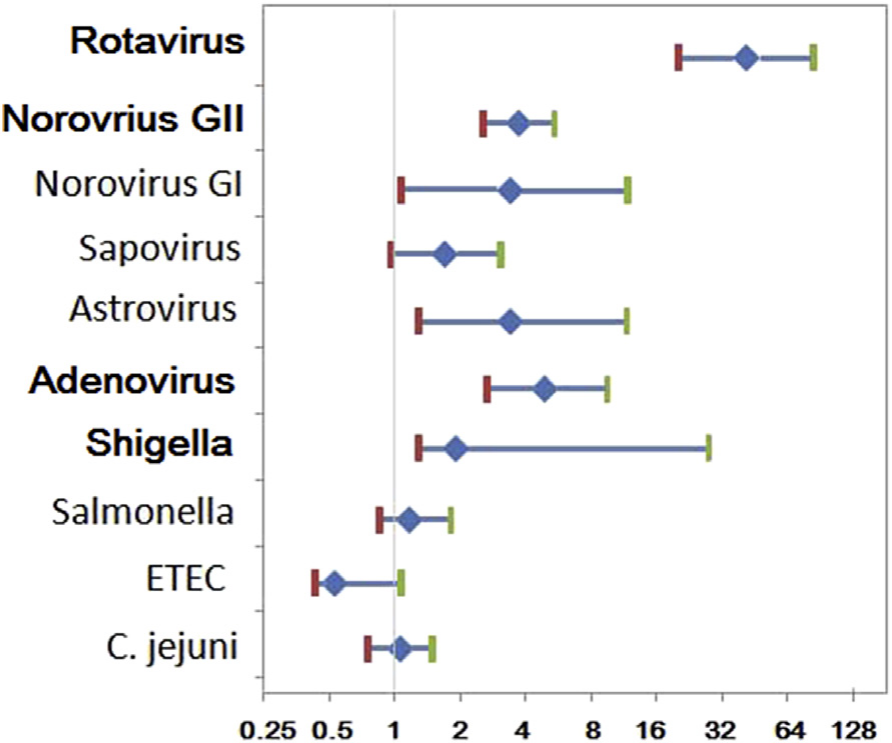
ORs of infections (using controls as reference category); 95% CIs indicated using standard error bars.

Ten pathogenic organisms—rotavirus, norovirus GII, *Shigella* spp., adenovirus, norovirus GI, astrovirus, sapovirus, *Salmonella* spp., ETEC and *C. jejuni*—that can cause diarrhoea were tested using qPCR. The data were analyzed qualitatively, describing whether any amount of the pathogen was detected or not. At least one enteric pathogen was detected in 377 (81.8%) of 461 children with diarrhoea compared to 215 controls (46.6%; p <0.001). Qualitatively, four pathogens—rotavirus, norovirus GII, *Shigella* and adenovirus—were significantly (p <0.01) associated with diarrhoea (Fig. 2, Table 3). Among these, rotavirus was found in 40.6% of cases and had the highest association with diarrhoea (OR 38.7, 95% confidence interval (CI) 18.8–78.7). Norovirus GII was found in 24.7% of cases and had an OR of 3.62 (95% CI 2.5–42.2). *Shigella* was identified in 18.6% of cases and had an OR of 1.79 (95% CI 1.2–2.6). Adenovirus was found in 10.9% of the cases and had an OR of 4.57 (95% CI 2.4–8.7) (Fig. 2). The other pathogens were found in small numbers (e.g. norovirus GI and astrovirus, each found in 14 case stools and four control stools) or were found in equal proportions in cases and controls (e.g. *Salmonella,* 63 case and 56 control stools or *C. jejuni,* 64 case and 64 control stools). The detection rates at the two sites (Hunan and Hebei) showed no significant differences for rotavirus, norovirus GII and GI, adenovirus, *Salmonella,* ETEC and *C. jejuni* in both case and control groups, but there were significant differences for *Shigella* and sapovirus in two sites in both case and control groups (p <0.01). More *Shigella* and sapovirus were found in Hebei province.

Incorporating a threshold for the effective concentration (the point above which the pathogen is considered likely to cause disease) categorizes individuals with very low levels with individuals with undetected levels of pathogen. The effective concentration is specific for each organism. For *Shigella,* the threshold has been estimated previously using receiver operating curve (ROC) analysis to be 10^4^ [14]; when applied to our current data, the OR was 5.1 and the 95% CI 1.1–23.6. ROC analysis was applied to rotavirus and norovirus GII measures; no other pathogens, including adenovirus, were detected in sufficient quantities to estimate the effective concentration from our data alone.

**Fig. 3:**
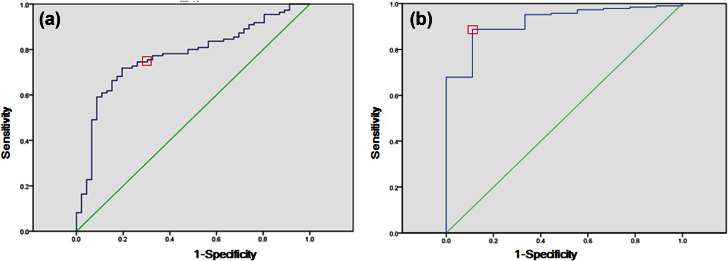
(a) ROC curve for model case–control status vs. qPCR for norovirus quantities. (b) ROC curve for model qPCR for rotavirus quantities. Curves were plotted by calculating sensitivity and 1-specificity of qPCR compared to case–control status. Squares indicate points on curve cutoff value.

ROCs for rotavirus and norovirus GII were constructed from the sensitivity and 1-specificity of the number of gene copies determined by qPCR (Fig. 3). Using diarrhoeal status as the outcome, the optimal cutoff value determined by the maximum Youden index was, for rotavirus, 72 copies per gram, with a sensitivity of 0.89 and a specificity of 0.89 (Fig. 3a). Using this cutoff value, 166 cases (36.0%) and only one control (0.2%) were identified as positive for rotavirus; the OR association with disease was 259.2 with a 95% CI of 36.1–1860. For norovirus GII, the maximum Youden index resulted in approximately 5840 copies of norovirus per gram, for a sensitivity of 0.69 and a specificity of 0.77 (Fig. 3b). With this proposed cutoff value, 84 cases (18.2%) and nine controls (2.0%) were identified as having a high level of norovirus GII in their stools (OR 10.6, 95% CI 5.45–42.2).

The attributable fraction can be used to describe the pathogen-specific burden for severe diarrhoeal disease. For rotavirus, the attributable fraction is 0.18, for norovirus GII 0.08 and for *Shigella* 0.01. The attributable fraction may also vary by age. Rotavirus was the only pathogen that differed across age groups in a statistically significant way (χ^2^ = 10, *df* = 3, p <0.017) (Table 4). Rotavirus had higher proportions of cases occurring in 13–24-month-olds (54%) and in 6–12-month-olds (38%) than either in the youngest (0–5 months, 10%) or oldest (>24 months, 7%) age categories (Table 4). The overall combined attributable fraction due to rotavirus, norovirus GII, adenovirus and *Shigella* was 0.52.

### Correlation between pathogens and clinical symptoms

Multiple pathogens were detected in 185 cases (40%) and 69 controls (15%). Thus, cases were significantly more likely to have multiple pathogens than controls (χ^2^ = 163, *df* = 4, p 10^−34^) (Table 4). To test whether there was a correlation between organisms in cases, we assumed a null hypothesis that each would occur at random with respect to each other. Two pairs were observed to have significant nonrandom distributions. Rotavirus and adenovirus were positively correlated (χ^2^ = 10, *df* = 1, p <0.002), and norovirus GII and *Salmonella* were positively correlated (χ^2^ = 23.8, *df* = 1, p 1 × 10^−6^).

In cases and controls, 73 instances (49.7%) of multiple pathogen identification involved rotavirus, 45 involved norovirus GII and only five involved *Shigella.* With so few instances, *Shigella* was not analyzed further. If a child has both rotavirus and any of the following sapovirus (p 0.003), astrovirus (p 0.046), adenovirus (p <0.0001), ETEC (p 0.002) or *C. jejuni* (p <0.0001), then that child was a case 100% of the time. If a child had norovirus GII and *Salmonella,* again, the child had diarrhoea 100% of the time (p 0.007). No other potential pathogen co-occurring with norovirus GII was statistically associated with being a case (Table 5).

Logistic regression found no correlation between coinfection and more severe clinical symptoms (including fever, vomiting and sick days).

## Discussion

In our survey of children with and without diarrhoea in rural China, a high proportion of cases (81.8%) were infected with at least one pathogenic organism, but a high proportion of controls were infected with at least one pathogen (49.5%) as well, albeit at a significantly lower rate. When the pathogen load was considered, we found that the OR of diarrhoea increased for rotavirus, norovirus GII and *Shigella* spp., indicating that these pathogens have an effective concentration above which they are likely to cause diarrhoeal symptoms. The population-attributable fraction was highest for rotavirus and then norovirus GII. Adenovirus was associated with diarrhoea by qualitative analyses, and none of the other six pathogens tested in this study was associated with diarrhoea. Thus, we found that enteric viruses have a greater positive association with paediatric diarrhoea than enteric bacteria, a finding similar to previous results [5], [6], [7], [8].

Among the cases in this study, rotavirus and norovirus GII were significantly correlated with diarrhoea. The attributable fraction was 0.18 and 0.08 respectively, as they accounted for 36 and 18% of the cases. Previous epidemiologic studies demonstrated that rotavirus and norovirus are leading causes of diarrhoea in young children in many countries, and most high-copy-number infections occurred among children younger than 2 years [7], [12]. Our data also show that the detection rate of these pathogens peaked between 6 and 12 months of age, after which they decreased. Because 72% of deaths associated with diarrhoea happen in the first 2 years of life, prevention and treatment of rotavirus and norovirus GII is crucial to lower the rate of diarrhoea-caused deaths among young children [5]. However, norovirus GII also had a high prevalence among our healthy controls (23.9%). The mere presence of norovirus is not necessarily enough to cause disease; the modeling work of Lopman *et al*. [24] suggested that if norovirus is a pathogen that confers partial immunity, then the prevalence of norovirus will be higher among controls in high-income countries than in low-income countries. Thus, it is doubly important to quantify the pathogen load in both symptomatic and healthy individuals when evaluating diarrhoea-causing agents in low-income countries, as we did with the ROC analysis to determine the effective concentration likely to cause disease. Also, it is more specific to ascribe an association between pathogens and diarrhoea in an individual sample with quantitative molecular thresholds [11]. In our study, we used a ROC to propose a cutoff value of 5840 copies per reaction of stool for norovirus GII, where above or below the designated cutoff value the OR differed sharply: 10.6 (above) vs. 0.9 (below). In contrast, the prevalence of rotavirus among healthy controls was low (1.7%), which supports estimates from previous studies [7]. When we estimated a cutoff value using an ROC, only one healthy control (0.2%) was identified as having a high level of rotavirus (OR 259). Calculating copy number cutoff values improves the distinctions between symptomatic and asymptomatic children even for pathogens present in low amounts in controls.

Previous studies have found that adenovirus, astrovirus and sapovirus are important causes of gastroenteritis in young children, with prevalences of 0.2 to 19% among children hospitalized for gastroenteritis [17], [22], [25]. We found similar prevalences for the three viruses (adenovirus 10.9%, astrovirus 3.0% and sapovirus 6.5%). Although a previous study found adenovirus, astrovirus and sapovirus primarily affect children under the age of 1 year [5], we found all three viruses in children between ages 1 and 5. Moreover, there were no significant differences among the prevalence of these viruses for the age groups. The prior work of Grant *et al*. [26] with American Indian children found that sapovirus and astrovirus did not occur in mixed infection. However, when we adjusted for pathogen load of rotavirus, we observed that when sapovirus, astrovirus and/or adenovirus co-occurred with rotavirus, 100% of the children had severe diarrhoea. A previous study in Kolkata, India, also showed a positive correlation between rotavirus and adenovirus among hospitalized patients [9].

Among the enteric bacteria, *Shigella* infections were the most common infections in this study and the only one associated with diarrhoea both qualitatively and quantitatively, which supports the results from prior studies [3], [7], [9], [14]. None of the other enteric bacteria was positively associated with diarrhoea in our population. The detection rate of ETEC and *C. jejuni* were similar in sick children (5.4 and 13.9% respectively) and healthy children (8.7 and 13.9% respectively), and logistic regression analysis failed to find a relationship between diarrhoea and either bacteria, even when taking copy numbers into account. However, when either of these bacteria co-occurs with rotavirus, 100% of the children have diarrhoea. In contrast, *Salmonella* did not occur with rotavirus but did co-occur with norovirus GII, and when it did, again, 100% of the children had diarrhoea. The cause of these nonrandom associations is not understood.

In one study, the authors found that norovirus interactions with enteric bacteria and human norovirus infection of B cells require the presence of histoblood group antigen (HBGA)-expressing enteric bacteria. Another study, performed at about the same time, found that bacterial microbiota fosters enteric virus persistence in a manner counteracted by specific components of the innate immune system [27]. It should be noted that HBGA has been identified as potential receptor or coreceptor of norovirus, and HBGA or other glycans have been suspected to be an important factor in rotavirus infection. It can thus be assumed that glycans carried by different enteric bacteria may play important an role in enteric virus infection [28], [29]. This might partly explain the specific bacteria co-occurrence with specific viruses, but details of the mechanisms or reasons need further study.

Our results suggest that symptomatic infections with enteropathogens in rural areas are not happenstance and asymptomatic infections are common, in China and worldwide [7], [10], [11], [12]. Our quantitative analyses increased the OR between the pathogen and diarrhoeal disease. As others before us, we found many instances of coinfection that occurred in nonrandom associations. We tested for ten potential pathogens which are the most common diarrhoeal pathogens. However, all ten combined had a total attributable fraction of less than one third, and although this number would be higher if we tested for the other known pathogens, we found the majority of diarrhoeal cases occur from unknown attributable causes.

## Transparency Declaration

Partly supported by the National Natural Science Foundation of China (grant 81290345) and by the Gates Foundation (grant OPP1016839). All authors report no conflicts of interest relevant to this article.

## Figures and Tables

**Table 1:** Primers and probes used in real-time PCR tests targeting RNA or DNA of diarrhoeagenic agents

Target pathogen	Target region	Primer/Probe	Primer/probe sequence (5′–3′)	Reference
GI	RdRp/capsid	Cog 1F	CGYTGGATGCGITTYCATGA	[15]
		Cog 1R	CTTAGACGCCATCATCATTYAC	
		Ring 1A	FAM-AGATYGCGATCYCCTGTCCA-BHQ1	
		Ring 1B	FAM-AGATCGCGGTCTCCTGTCCA-BHQ1	
GII	RdRp/capsid	Cog 2F	CARGARBCNATGTTYAGRTGGATGAG	[15]
		Cog 2R	TCGACGCCATCTTCATTCACA	
		Ring 2	JOE-TGGGAGGGCGATCGCAATCT-BHQ1	
Sapovirus	RdRp/capsid	CU-SV-F1	GACCAGGCTCTCGCYACCTAC	[16]
		CU-SV-F2	TTGGCCCTCGCCACCTAC	
		CU-SV-R	CCCTCCATYTCAAACACTAWTTTG	
		CU-SV-Probe	FAM-TGGTTYATAGGYGGTAC-BHQ1	
Rotavirus	NSP3	ROTA-F	ACCATCTACACATGACCCTC	[22]
		ROTA-R	GGTCACATAACGCCCC	
		ROTA-P	FAM- ATGAGCACAATAGTTAAAAGCTAACACTGTCAA-TAMRA	
Astrovirus	ORF1a	AS-F	TCTYATAGACCGYATTATTGG	[18]
		AS-R	TCAAAATTCTACATCATCACCAA	
		AS-P	FAM-CCCCADCCATCATCATCTTCATCA-BQ1	
Adenovirus	hexon	AdV-F	GCCCCAGTGGTCTTACATGCACATC	[18]
		AdV-R	GCCACGGTGGGGTTTCTAAACTT	
		AdV-Probe	FAM-TGCACCAGACCCGGGCTCAGGTACTCCGA-TAMTRA	
*Campylobacter jejuni*	ORF-C	82F	TTGGTATGGCTATAGGAACTCTTATAGCT	[19]
		197R	CACACCTGAAGTATGAAGTGGTCTAAGT	
ETEC	*elt* A	ETEC_LT_F1	GGCGACAAATTATACCGTGC	This study
		ETEC_LT_R1	TGTGTTCCTCTCGCGTGATC	
Shigella	*ipa* H	Shig_ipaH_F	CGGAATCCGGAGGTATTGC GGTATTGC	[20]
		Shig_ipaH_R	CCTTTTCCGCGTTCCTTGA	
Salmonella	ttrC/ttrA	ttr-6	CTCACCAGGAGATTACAACATGG	[21]
		ttr-4	AGCTCAGACCAAAAGTGACCATC	
		ttr-5 (probe)	FAM-CACCGACGGCGAGACCGACTTT-BHQ1	

BHQ1, black hole quencher 1; ETEC, enterotoxigenic *Escherichia coli;* FAM, 6-carboxyfluorescein; JOE, 6-carboxy-4,5-dichloro-2,7-dimethyoxyfluorescein.; TAMRA, 6-carboxytetramethylrhodamine.

**Table 2:** Characteristics of patients with diarrhoea and their matched control in Hunan and Hebei, China, May 2011 to January 2013

Characteristic	Cases (*n* = 461), *n* (%)	Controls (*n* = 461), *n* (%)
Age group		
0–6 months	125 (27%)	112 (24%)
6–11 months	177 (38%)	179 (39%)
12–24 months	132 (29%)	140 (30%)
24–36 months	17 (4%)	20 (4%)
36–59 months	10 (2%)	10 (2%)
Sex		
Male	300 (65%)	297 (64%)
Female	161 (35%)	164 (36%)
Location		
Hunan	222 (48%)	222 (48%)
Hebei	239 (52%)	239 (52%)
No. pathogens		
0	84 (18%)	246 (53%)
1	192 (42%)	146 (32%)
2	115 (25%)	58 (13%)
3	53 (11%)	8 (2%)
4	17 (3%)	3 (0%)

**Table 3:** Comparison between 461 patients and 461 controls with respect to real-time PCR detection rates and copy numbers in Hunan and Hebei, China

Pathogen	Cases, *n* (%)^a^			Controls, *n* (%)^a^			p	Cases, mean (SD)	Controls, mean (SD)
Total	Hunan	Hebei	Total	Hunan	Hebei	Total	Total
Rotavirus	187 (40.6)	99	88	8 (1.7)	4	4	<0.001	62 748 (361 729)	5 (90)
Norovirus GII	114 (24.7)	59	55	40 (8.7)	15	25	<0.001	881 209 (6 953 733)	32 385 (497 573)
Norovirus GI	14 (3.0)	11	3	4 (0.9)	2	2	0.017	29 321 (536 789)	8866 (180 451)
Sapovirus	30 (6.5)	7^b^	23^b^	19 (4.1)	4^b^	15^b^	0.106	132 885 (1 489 731)	12 938 (200 747)
Astrovirus	14 (3.0)	1^b^	13^b^	4 (0.9)	4	0	0.017	88 775 582 (1 900 024 087)	42 963 (907 251)
Adenovirus	50 (10.9)	17	33	12 (2.6)	5	7	<0.001	2 150 466 (20 808 127)	9300 (154 549)
*Shigella*	86 (18.6)	20^b^	66^b^	53 (11.5)	17^b^	36^b^	0.006	18 870 (396 865)	1453 (30 749)
*Salmonella*	63 (13.7)	29	34	56 (12.1)	41^b^	15^b^	0.49	240 (3497)	6 (75)
ETEC	25 (5.4)	10	15	40 (8.7)	14	26	0.054	1028 (18 149)	1205 (17 925)
*Campylobacter jejuni*	64 (13.9)	38	26	64 (13.9)	39	25	>0.5	1728 (22 127)	37 (599)

ETEC, enterotoxigenic *Escherichia coli.*

a Presence of pathogen based on detection of any amount by PCR.

b Significant difference (p <0.01) between Hunan and Hebei province by chi-square test.

**Table 4:** Prevalence of four main infectious aetiologies among diarrhoeal and healthy children, by age groups, China, May 2011 to January 2012

Infectious agent	0–6 months		7–12 months		13–24 months		>24 months	
Case (*n* = 125)	Control (*n* = 125)	Case (*n* = 177)	Control (*n* = 177)	Case (*n* = 132)	Control (*n* = 132)	Case (*n* = 27)	Control (*n* = 27)
Rotavirus	26 (10.4%)	0 (0)	67 (37.9%)	1 (0.6%)	71 (53.8%)	0 (0)	2 (7.4%)	0 (0)
Norovirus GII	17 (13.6%)	2 (1.6%)	36 (20.3%)	2 (1.1%)	23 (17.4%)	4 (3.0%)	3 (11.1%)	0 (0)
Adenovirus	13 (10.4%)	2 (1.6%)	16 (9.0%)	6 (3.4%)	17 (12.9%)	4 (3.0%)	4 (14.8%)	0 (0)
*Shigella*	2 (1.6%)	0 (0)	1 (0.6%)	0 (0)	0 (0)	0 (0)	5 (18.5%)	1 (3.7%)

**Table 5:** Coinfection of rotavirus, norovirus or *Shigella* and other pathogens among diarrhoeal and healthy children, China, May 2011 to January 2012

Children	Rotavirus	Norovirus GII	*Shigella*
Control			
Any coinfection	1	8	0
Norovirus GII	1	—	0
Norovirus GI	1	8	0
Sapovirus	0	0	0
Astrovirus	0	1	0
Adenovirus	0	0	0
*Shigella*	0	2	—
*Salmonella*	0	0	0
ETEC	0	0	0
*Campylobacter jejuni*	0	3	0
Cases			
Any coinfection	72	37	5
Norovirus GII	5	—	0
Norovirus GI	6	3	0
Sapovirus	13	5	0
Astrovirus	6	3	0
Adenovirus	29	4	2
*Shigella*	0	0	—
*Salmonella*	0	11	2
ETEC	15	4	2
*Campylobacter jejuni*	24	13	0

ETEC, enterotoxigenic *Escherichia coli.*

Stools considered positive for rotavirus, norovirus GII and *Shigella* if quantitative PCR amount exceeded threshold. All other pathogens were considered positive if any amount was detected.
